# Medication discrepancies across multiple care transitions: A retrospective longitudinal cohort study in Italy

**DOI:** 10.1371/journal.pone.0191028

**Published:** 2018-01-12

**Authors:** Marco Bonaudo, Maria Martorana, Valerio Dimonte, Alessandra D’Alfonso, Giulio Fornero, Gianfranco Politano, Maria Michela Gianino

**Affiliations:** 1 Department of Public Health Sciences and Pediatrics, Università di Torino, Torino, Italy; 2 Public Health Authority, ASLTO2, Torino, Italy; 3 AOU Città della salute e della Scienza, Teaching Hospital, Torino, Italy; 4 Department of Control and Computer Engineering, Politecnico di Torino, Torino, Italy; TNO, NETHERLANDS

## Abstract

**Purpose:**

Medication discrepancies are defined as unexplained differences among regimens across different sites of care. The problem of medication discrepancies that occur during the entire care pathway from hospital admission to a local care setting discharge (namely all types of settings dedicated to formal care other than hospitals) has received little attention in the medical literature.

The present study aims to (1) determine the prevalence of medication discrepancies that occur during the entire care pathway from hospital admission to local care setting discharge, (2) describe the discrepancy and medication type, and (3) identify potential risk factors for experiencing medication discrepancies in patient care transitions. Evidence from an integrated health care system, such as the Italian one, may explain results from other studies in different healthcare systems.

**Methods:**

A retrospective longitudinal cohort study of patients admitted from July 2015 to July 2016 to the Giovanni Bosco Hospital serving Turin, Italy and its surrounding territory was performed. Discrepancies were recorded at the following four care transitions: T1: Hospital admission; T2: Hospital discharge; T3: Admission into local care settings; T4: Discharge from local care settings. All evaluations were based on documented regimens and were performed by a team (doctor, nurse and pharmacists).

**Results:**

Of 366 included patients, 25.68% had at least one discrepancy. The most frequent type of discrepancy was from medication omission (N = 74; 71.15%). Only discharge from a long-stay care setting (T4) was significantly associated with the onset of discrepancies (p = 0.045). When considering a lack of adequate documentation, not as missing data but as a discrepancy, 43.72% of patients had at least one discrepancy.

**Conclusions:**

This study suggests that an integrated health care system, such as Italian system, may influence the prevalence of discrepancies, thus highlighting the need for structured multidisciplinary and, if possible, computerized medication reconciliation to prevent medication discrepancies and improve the quality of medical documentation.

## Introduction

Medication discrepancies are defined as unexplained differences among regimens across different sites of care [1]. They are categorized into documented and undocumented discrepancies, the latter of which are further subdivided into intentional or unintentional.

Recent scientific evidence considers medication discrepancies to be an important public health problem with clinical and economic consequences [2–4]. Indeed, discrepancies can be harmful, leading to inadequate prescriptions, interruptions of treatment, adverse drug events, longer hospital stays and an increase in hospital readmissions and emergency room visits, which are expensive for the health care system [2–7]. Several studies have identified care transitions as time points with a greater risk of drug discrepancies [8–10]. Additionally, medication discrepancies in care transitions could occur more easily in the absence of a formalized system for medication reconciliation and with poor quality of communication between primary and secondary care [2].

Medication reconciliation is a formal process that involves matching the medicines that the patient *should* be prescribed with those that are *actually* prescribed and involves adequately reporting any therapy change [3,11]. Medication reconciliation has been identified in the literature as an important tool for preventing pharmacological discrepancies and the World Health Organization, in the “*Action on Patient Safety”*, considers medication reconciliation to be one of the five top strategies for ensuring patient safety [12]. Additionally, the Joint Commission on Accreditation of Healthcare Organizations included medication reconciliation in the accreditation standards [13]. The problem of medication discrepancies that occur during the entire care pathway from hospital admission to a local care setting discharge (namely, all types of settings dedicated to formal care other than hospitals) has received little attention in the medical literature. Indeed, there are studies in the literature on single points of transitions of care: most analyze discrepancies upon hospital admission or discharge [3,4,14–18], and little is known about the prevalence of medication discrepancies upon admission or discharge from local care settings, such as the home and rehabilitation facilities [1,19]. According to the literature unintended medication discrepancies occurred in 25%-70% of hospital admissions and in 33%-96% of hospital discharges [4,6,20–22] and up to 71% of patients admitted to local care settings [1,19].

Current evidence is limited in Italy, and there are no studies conducted in a local care setting [11].

New evidence may be important from an integrated health care system, such as the Italian health system, and may help to explain results from other studies in other countries with different healthcare systems. Italy’s healthcare system is a regionally organized national health service (Servizio Sanitario Nazionale, SSN) that has two underlying principles. First, every Italian citizen and foreign resident has the right to healthcare, and second, the system covers all necessary treatments. Local health authorities (ASL) are responsible for the management of all health services in their area, from prevention to care in hospitals and in any type of setting dedicated to formal care other than hospital care and located in the districts. Italy’s healthcare system has made considerable efforts to achieve co-ordination and integration of care, which allows the patient to be followed throughout his/her care pathway. For this aim, one unit in hospitals (NOCC) and one in the district (NDCC) that oversee all transitions of care to manage the most complex medical cases and to ensure the admission to and discharge from a health-care setting were introduced. With Recommendation 17 [23], the Ministry expects that during this pathway attention will also be devoted to pharmacological reconciliation.

The present study aims to (1) determine the prevalence of medication discrepancies that occur during the entire care pathway from hospital admission to a local care setting discharge, (2) describe the type of discrepancy and type of medication, and (3) identify potential risk factors for experiencing medication discrepancies during patient care transitions.

## Methods

A retrospective longitudinal cohort study of patients admitted to the Giovanni Bosco Hospital which serves Turin, Italy and its surrounding territory was performed. The following four local care settings: (i) Long-stay Care, (ii) Rehabilitation, (iii) Supported discharge in multiple facilities and (iv) Integrated Home Care (ADI), were studied. Giovanni Bosco Hospital is a hub urban hospital in Turin in Local Health Authority ASL TO2, with more than 3400 health workers and approximately 330 beds and 11,000 discharges/year. It covers an area of over 420,000 inhabitants. Local care setting were positioned in Turin city and on its surrounding territory [24,25]. The study included all patients admitted to the Giovanni Bosco Hospital from July 2015 to July 2016 who met the following inclusion criteria: (i) age > 18 years, (ii) discharged from one of the four hospital wards (internal medicine, geriatrics, neurology, and orthopedics), (iii) admitted to and discharged from one of the four local care settings indicated above, and (iv) beginning and closing their clinical pathway during the above-cited period.

Discrepancies, defined as any undocumented and unintentional incongruity between the documented sources that could result in therapy errors [11,14], were recorded for the following four care transitions: T1: hospital admission; T2: hospital discharge; T3: admission to local care setting; T4: discharge from local care setting. Medication discrepancies were identified using a structured tool that retraces the medication reconciliation process and uses the same variables and sources of documented regimens defined by Ministero della Salute Recommendation [23]. Supported by the above cited recommendation, this analysis was conducted by a team of all professional categories involved in medication treatment (a medical doctor, a nurse and two clinical pharmacists, able to evaluate if the decided treatments were according to the patient clinical pathway) in the following way. For T1, the emergency department (ED) list recorded on the ED form was compared to the first prescriptions in the hospital ward that were recorded in the chart. For T2, the prescriptions on the last hospitalization day recorded on the chart were compared to the therapy list in the discharge letter. For T3, the prescriptions in the discharge letter were compared to the first prescriptions in the local care setting that were recorded on the chart. For T4, the prescription from the last care day were compared to the prescriptions recorded in the discharge letter. Evaluation of medication discrepancies was performed by the team. The doctor and the nurse, separately, analyzed transitional documentation and reported all discrepancies detected, and all the discrepancies were subjected to the pharmacists’ evaluation. Then, by re-evaluating the patients’ clinical history, the team defined whether discrepancies were in line with the care pathway (intentional) or whether they were the result of a clinical error (unintentional). In more complicated cases the doctor who had treated the patient was consulted. Only, the unintentional discrepancies were recorded. Discrepancies were classified as omissions of medication; errors/omissions of the drug name, dose, dosage, frequency and administration type; duplications; and interactions. The drugs involved in the discrepancies were categorized by their mechanisms of action. This study termed “not evaluable discrepancies” as the following: i) the patient was admitted to the hospital ward immediately without transitioning to the ED; and ii) there was no discharge letter because the patient died or was transferred to the ED from a local care setting. The “not evaluable discrepancies” were always considered to be missing.

This study defined “lack of documentation” as any situation in which there was no trace of a clinical document. As the “lack of a documentation” did not allow to assess the presence or absence of discrepancies, we evaluated two possible hypotheses. In the first hypothesis, assuming that “lack of documentation” was a “not evaluable discrepancies” was considered to be missing; in the second hypothesis, “lack of documentation” was considered to be a discrepancy with the aim of considering the worst possible scenario.

### Statistical analysis

To automate statistical analysis, we developed a general and robust R [26] pipeline that processed the input data through the following steps:

#### Data sources

The input data were presented to the pipeline as a csv file in which each row represented a patient along with his or her drug discrepancies. Given this format, multiple rows belonging to the same patient were allowed (e.g., a single patient might be discrepant for more than one drug).

Data matrix columns contained several attributes used for stratification: personal attributes (e.g., gender, age, nationality) and discrepancy attributes (e.g., missing or erroneously administered drugs) detailed for each transition of care. Moreover, during the preprocessing phase, raw attributes have been combined into functions that allowed to compute derived stratification values (e.g., the difference between hospital admission and discharge dates has been used to compute the number of days a person has been hospitalized), which in turns resulted into more meaningful data for further analysis steps.

#### Descriptive analysis

Preprocessed data have been analyzed in terms of cumulative analysis and descriptive analysis. In the cumulative analysis we computed the global discrepancy assessment, by taking into account multiple discrepancies (when available) for every single patient, which resulted in 398 rows, one for every combination of a patient and a specific discrepancy. In the latter analysis, we instead resorted to a patient-centric analysis. We therefore merged multiple discrepancies of a single patient in a single row, thus reducing the database to 366 rows, one for each patient.

The pipeline produced as output two files as follows: i) cumulative analysis, and, ii) descriptive analysis. The first file, cumulative analysis, contained for each transition of care the stratification in terms of categories. The figures in the output file were expressed as cumulative value and relative percentage over the totals. In the descriptive analysis output file, all the database columns of interest were analyzed according to their nature. Categorical variables have been stratified, while numerical variables have been analyzed in terms of statistical distribution computing their mean, standard deviation and quartile distribution. Some numerical variables that allowed for further categorization (e.g., age) have also been factorized into groups (i.e., age groups) in order to simplify the following odds ratio analysis. Moreover, patients with “lack of documentation” have been analyzed in two different hypotheses depicted above. To obtain more consistent and conservative measures "not evaluable discrepancies" patients have been programmatically removed.

#### Stratification & risk analysis

After the descriptive analysis, the pipeline automatically computed a confusion matrix for each categorical variable in order to perform significance tests, and data were prepared in a convenient fashion for further odds ratio analysis. Confusion matrices were then corrected by evaluating numerosity constraints and discarding low abundance strata (fewer than 8 elements) in order to reach sufficient significance in the statistical tests. Both the Chi-Square test (high numerosity strata) and Fisher exact test (low numerosity strata) were performed. For computing the drug discrepancy odds ratio for each stratum, we defined a control value to be used as a reference for each confusion matrix, and we used the strata with the lowest marginal probability as a control (i.e., with odds ratio = 1) and mapped the other strata against this one, guaranteeing that all of the other odds ratios were greater than 1. An output file summarizes every stratification in terms of its odds ratio along with 95% confidence intervals and the p-value of both the chi-square and Fisher’s exact tests.

The study was approved by Ethics Committee of the Turin territory, Prot. N. 0012774, August 1, 2016. An informed written consent was obtained in agreement with Authorization on processing personal data GU n. 302, December 27, 2013. The patients’ private health information (PHI) was ensured by the de-identification of data.

## Results

Between July 2015 and July 2016, 366 patients met the inclusion criteria and were included in the study. The mean (SD) age of the patients was 80.77 (10.66) years, and 57.92% were female. Table 1 lists the patient characteristics.

**Table 1:** Patients’ characteristics (N = patients).

		N = 366	%
**Sex**	Female	212	57.92
**Age**	<=65	35	9.56
	66–84	200	54.64
	>=85	131	35.79
**Nationality**	Italian	359	98.09
	Romanian	3	0.82
	Non -EU*	4	1.08
**Pathology**	Fractures and orthopedic implant complications	91	24.86
	Vertebral-cerebral vasculopathy and infarct / intracranial hemorrhages	41	11.20
	Cardiovascular diseases	34	9.29
	Respiratory tract infections	23	6.28
	Dehydration, heat effects, hydro-electrolyte imbalance	18	4.92
	Dementia and delirium	17	4.64
	Solid tumors	15	4.10
	Septicemia	15	4.10
	Other**	112	30.67

*Non-EU includes the following nationalities: Nigerian, Chinese, Ethiopian, Moroccan. Each nationality has a unit value (N = 1; 0.27%)

**The "”other"” category includes the followings:

Osteoarthritis and cervical spondylosis, asthma and respiratory failure (n = 14; 3.83%); urinary tract infections (n = 8; 19.2%); pulmonary embolism, gastrointestinal infections, nephrotic syndrome, genitourinary diseases and renal failure (n = 6; 1.64%); contusions, intracranial trauma, gastritis and gastroduodenal ulcers (n = 4; 9.1%); anemia, depression and other mental disorders, hemiplegia, hemiparesis, hepatitis, hepatic encephalopathy, unspecified infection, esophageal disease, rhabdomyolysis, lymphatic and hematological tumors, and pressure ulcers (n = 3; 0.82%); bowel obstruction, gastrointestinal bleeding, and syncope (n = 2; 0.55%); ascites, diabetes, rashes, nose and throat infections, central nervous system infections, skin infections, neuropathy, muscle disease, abdominal aortic aneurysm, syphilis, thyroiditis, and pleural effusion (n = 1; 0.27%); and missing diagnoses (n = 5; 1.37%).

Most (93.72%, N = 343) of the enrolled patients were from the emergency department, while 6.28% (N = 23) had a planned hospital admission.

**Fig 1:**
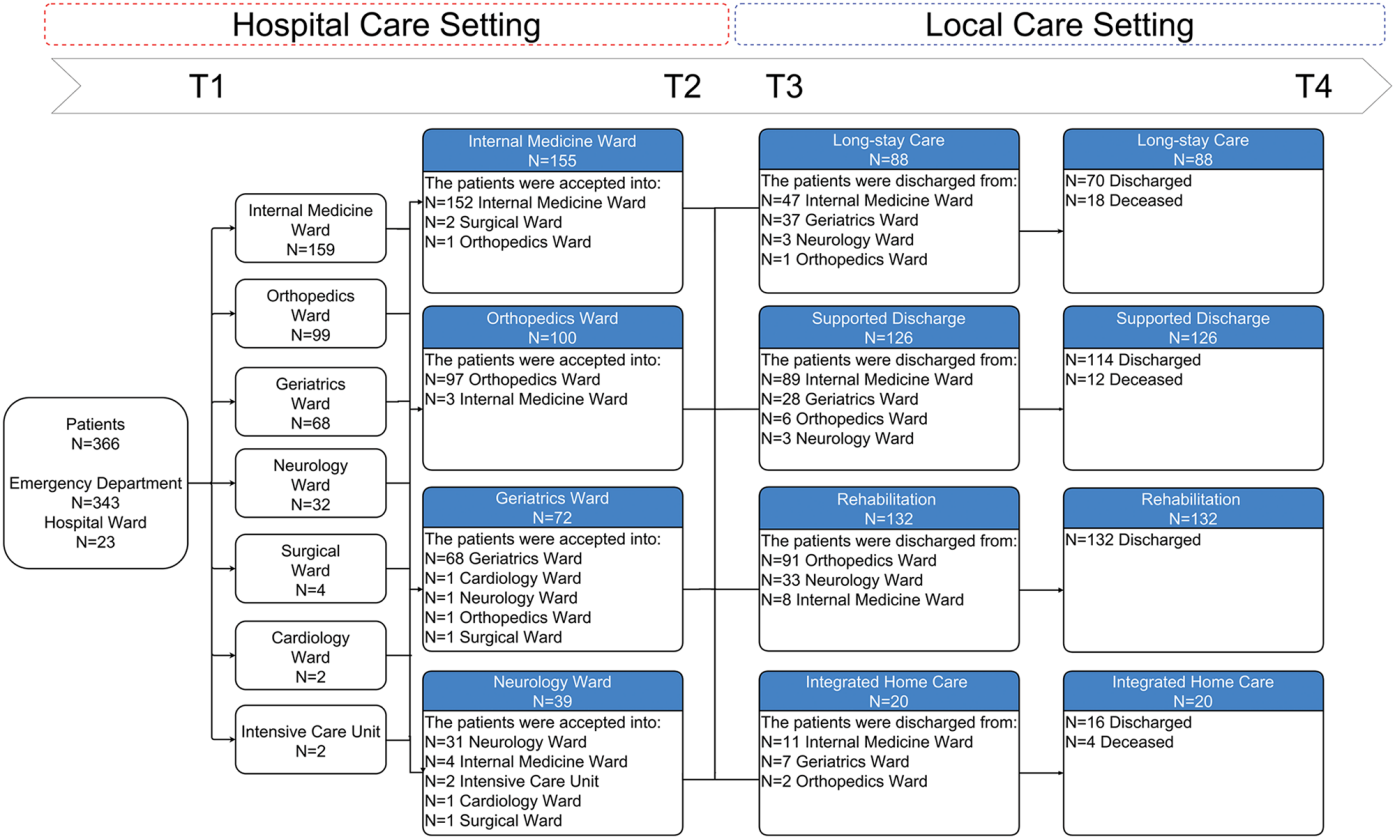
Number of patients sampled in the care pathway through health facilities. T1: Hospital admission T2: Hospital discharge T3: Admission into local care setting T4: Discharge from local care setting.

Of the participants, 43.44% were admitted to internal medicine, 27.05% to orthopedics, 18.58% to geriatrics, 8.74% to neurology, 1.09% to surgery, 0.55% to cardiology and 0.55% to the intensive care unit. The patients’ hospital discharge rates were 42.35% from internal medicine, 27.32% from orthopedics, 19.67% from geriatrics and 10.66% from neurology. Additionally, 36.06% of patients were admitted to rehabilitation settings, 34.43% to supported discharge settings, 24.04% to long-stay care and 5.46% to integrated home care (ADI) (Fig 1).

### First hypothesis: “lack of documentation” was considered to be missing

Considering the first hypothesis, the total number of discrepancies was 104; pharmacological discrepancies involved 94 patients, so 25.68% of the total number of patients had at least one discrepancy. The most frequent type of discrepancy was omission of medication (N = 74; 71.15%) (see Table 2).

**Table 2:** Type of medication discrepancy (N = discrepancies).

	First Hypothesis (N = 104)		Second Hypothesis (N = 218)	
	N	%	N	%
**Omission of medication**	74	71.15	74	33.94
**Error / omission of medication name**	9	8.65	9	4.13
**Error / omission of medication dose**	11	10.58	11	5.05
**Error / omission of drug dose**	10	9.62	10	4.59
**Lack of documentation**	-	-	114	52.29

Furthermore, the most frequent category of drugs involved in the discrepancy was “proton pump inhibitors” (N = 11; 11.34%) (see Table 3).

**Table 3:** Drug categories involved in at least one discrepancy, first hypothesis (N = frequencies).

	N = 97	%
**Proton pump inhibitors**	11	11.34
**Diuretics, ACE inhibitors and angiotensin receptor blockers**	10	10.31
**Antithrombotics**	9	9.28
**Folic acid**	6	6.19
**Beta-blockers**	6	6.19
**Hyperuricemia reducers**	6	6.19
**Anxiolytics and antidepressants**	5	5.15
**Antiepileptics**	5	5.15
**Statins**	5	5.15
Other*		

* The “other” category includes the followings: alpha blockers and 5-alpha reductase inhibitors, thyroid hormone, and steroids (n = 4; 4.12%); bronchodilators (n = 3; 3.09%); antihypertensive REC alpha, calcium and vitamin D, iron, prepared glaucoma and mitotic (n = 2; 2.6%); and painkiller, antidopaminergics, anti lithogenics, antimalarials, antiparkinsonians, antivirals, acetyl-cholinesterase inhibitors, hypoglycemic, laxatives, nitrates, and psychostimulants (n = 1, 1.3%)

Table 4 shows the association between the presence of 1 or more pharmacological discrepancies in each transition and the independent variables (see Statistical Analysis). In this first hypotheses, no variables were significantly associated with discrepancies at the first, second and third transition (Table 4). By contrast, only T4 associations were significant: discharge from a specific local care setting was associated with a discrepancy (p = 0.045), and the length of stay was a statistically significant variable (p<0.001). Gender also appears to be associated with drug discrepancies upon the fourth transition (p = 0.009).

**Table 4:** P-value of the chi square test (N = patients).

	Hypothesis 1				Hypothesis 2			
Variable	T1 (N = 308)	T2 (N = 366)	T3 (N = 317)	T4 (N = 298)	T1* (N = 329)	T2* (N = 366)	T3* (N = 356)	T4* (N = 332)
**Age**	p = 0.856	p = 0.796	p = 0.217	p = 0.411	p = 0.481	p = 0.799	p = 0.296	p = 0.27
**Gender**	p = 0.855	p = 0.84	p = 0.139	*p = 0*.*009*	p = 0.66	p = 0.846	p = 0.767	*p = 0*.*049*
**Length of stay (days) in Hospital ward/Local care setting**	―	p = 0.493	―	*p<0*.*001*	―	p = 0.499	―	*p = 0*.*001*
**Changing ward Assignment**	―	p = 1	―	―	―	p = 1	―	―
**Hospital ward admission**	p = 0.118	―	―	*―*	p = 0.188	―	―	―
**Hospital ward discharge**	―	p = 0.544	p = 0.119	―	―	p = 0.547	*p = 0*.*001*	―
**Local care setting admission**	―	―	p = 0.374	―	―	―	*p<0*.*001*	
**Local care setting discharge**	―	―	―	*p = 0*.*045*	―			p = 0.23

*Second Hypothesis

Finally, Table 5 shows the odds ratios at care transitions. Considering the first hypothesis, the following three variables were associated with a higher risk of discrepancies for the patients: long-stay care setting discharge [OR 2.59 (1.042–6.464)], and length of stay of or less than 10 days [OR 11.43 (4.263–30.639)] and between 11 and 30 days [OR 3.72 (1.532–9.037)] at T4.

**Table 5:** Risk profile at care transitions, odds ratio (95% CI).

	Hypothesis 1		Hypothesis 2	
	T3	T4	T3*	T4*
**Gender**^ǂ^				
M	2.73 (0.804–9.257)	2.63 (1.239–5.561)	1.12 (0.617–2.035)	1.74 (1.016–2.988)
F	1 (0.246–4.062)	1 (0.436–2.293)	1 (0.569–1.758)	1 (0.58–1.723)
**Length of stay (days) in local care setting**				
≤10	―	11.43 (4.263–30.639)	―	4.69 (2.015–10.923)
11–30	―	3.72 (1.532–9.037)	―	2.16 (1.119–4.185)
≥31	―	1 (0.453–2.209)	―	1 (0.613–1.631)
**Local care setting admission**				
Integrated home care	―	―	17.36 (3.942–76.416)	―
Rehabilitation	1 (0.138–7.229)	―	2.57 (1.056–6.261)	―
Supported discharge	2.89 (0.571–14.647)	―	1.45 (0.559–3.745)	―
Long-stay care	1.33 (0.184–9.667)	―	1 (0.336–2.98)	―
**Hospital ward discharge**				
Internal medicine	1 (0.245–4.079)	―	1.13 (0.306–4.181)	―
Orthopedics		―	4.11 (1.163–14.522)	―
Geriatrics	3.57 (0.971–13.119)	―	2.07 (0.533–8.026)	―
Neurology	1.98 (0.338–10.883)	―	1 (0.189–5.289)	―
**Local care setting discharge**				
Integrated home care	―	―	―	―
Rehabilitation	―	1.04 (0.414–2.614)	―	1 (0.495–2.02)
Supported discharge	―	1 (0.381–2.627)	―	1.11 (0.542–2.271)
Long-stay Care	―	2.59 (1.042–6.464)	―	1.88 (0.88–3.962)

*Second Hypothesis

^ǂ^ F: Female; M: Male

### Second hypothesis: “lack of documentation” was considered a discrepancy

Considering the second hypothesis, the total number of discrepancies was 218; 114 were from a lack of documentation. The pharmacological discrepancies involved 160 patients, so 43.72% had at least one discrepancy. Table 4 shows that no variables were significantly associated with discrepancies at T1 and T2 (Table 4). By contrast, the T3 and T4 associations were significant.

The hospital ward discharge and the local care setting admission in the third transition were associated with discrepancies (p = 0.001 and p<0.001, respectively). Finally, the length of stay and gender were significantly correlated with pharmacological discrepancies in the fourth transition (p = 0.001 and p = 0.049, respectively).

Table 5 shows that integrated home care admission [OR 17 (3.942–76.416)] and patients discharged from orthopedic ward [OR 4 (1.163–14.522)] had higher odds of discrepancy compared to long stay care settings and patients discharged from neurology respectively in T3. Finally, a length of stay of or less than 10 days and between 11 and 30 days was associated with an increased risk of discrepancies [OR 4.69 (2.015–10.932); OR 2.16 (1.119–4.185) respectively] in the fourth transition.

## Discussion

To our knowledge this study is the first to consider the complete pathway for patients from hospital admission to local care setting discharge. The literature shows that studies on medication reconciliation have focused on a single point of care transition.

Our study demonstrates that 25.68% and 43.72% of patients had at least one medication discrepancy in the first and second hypotheses, respectively. This rate of medication discrepancy is significant because it concerns the medication discrepancies during the entire care pathway from hospital admission to a local care setting discharge and it is lower than that from most of the reviewed studies, which focused on one transition care, especially in hospital transitions. Indeed, the reported discrepancies, involved 25%-70% of hospital admissions and 33%-96% at hospital discharge [4,6,20–22]. Our results may be influenced by the fact that the Italian healthcare system is set up to manage the continuity of care and to reduce fragmentation. The use of units (NOCC; NDCC) that facilitate the transition from hospital care to local care settings along with the presence of structured documentation upon some transitions and the participation of pharmacists, play in favor of low drug discrepancies. This suggestion justifies the best result compared to that reported from other countries with a different healthcare system such as the US [22,27] and Canada [6,20].

Previous studies have shown that the omission of a medication [6] and incomplete prescriptions [20] were the most common discrepancies. These findings are consistent with those of our study, which reported the omission of medication as the most frequent type of discrepancy. Our findings highlight that the variables associated with the risk of discrepancy change from hypothesis 1 to hypothesis 2. Considering “lack of documentation” as missing (hypothesis 1), there is only a statistically significant association at the fourth transition, particularly between the local care setting discharge and the onset of discrepancies. Moreover, our findings show that a long-stay care discharge has an increased risk of drug discrepancy. This result may be explained by the multiple organizations of care settings and their critical issues of work environment [28], by the high number of drugs, and by the frequent lack of standardized procedures for pharmacological reconciliation in this setting. This observation is consistent with those of previous studies that underlined the importance of a drug reconciliation module that keeps track of the changes in therapy for patients who are discharged to long-stay care settings [22,29,30].

Considering the second hypothesis, both hospital ward discharges and local care setting admissions at the third transition are significantly associated with the onset of discrepancies. Our results demonstrate a higher risk of discrepancy upon discharge from the orthopedic ward. This result may be attributed to the observation that this ward did not report a list of drugs in the discharge letter; instead the therapy sheet was included in the clinical chart, which should be sent to the local care setting. However, this practice was not effective because the therapy sheet was frequently not sent during the care transition. Consistent with the findings of Monfort et al., who presented similar results in which the discharge prescriptions from an orthopedic ward were often incomplete or the transmission of medication information at discharge was often lacking [31], we suggest the use of a structured medication reconciliation form. Considering again the second hypothesis, at the third transition, the local care setting admission is associated with medication discrepancies. Admission to an Integrated home care carries the highest risk of discrepancy. This result may be affected by the poorly structured or missing documentation. As reported in the study by Cua et al., documentation of medication changes, their rationale, and whether changes are temporary or permanent are often lacking [32]. A systematic review conducted by Tam et al., stressed that the prevailing pharmacological discrepancy in the home situation is the lack of a drug list [17]. Furthermore, a 2009 review conducted by Bayoumi et al. in primary care demonstrated that the lack of quality information impacts medication reconciliation [33]. The documents in the home situation may have disadvantages of bad handwriting, delays in preparation and missing or incorrect information, increasing the risk of drug discrepancies [34].

A noteworthy result is the risk of drug discrepancy in relation to the length of stay in local care settings, which indicates that there is a higher risk of discrepancy for a ≤10 days length of stay than for longer periods. Comparison of this result with those of other studies are negatively affected by variations in the point of transition. Indeed, few studies in the literature that evaluate the correlation between the length of stay and risk of drug discrepancies focus on the length of stay in a hospital and do not evaluate the local care setting. One study indicates that the possible occurrence of discrepancies is 1.70-fold greater for patients who remain hospitalized for a longer time, but this result is not significant (p = 0.065) [22]. An explanation for a lower risk with a longer stay could be that spending more time in the local settings could improve the understanding of the patient’s condition and result in more attention to his or her therapy.

The lack of communication among professional workers upon care transitions, identified as “lack of documentation”, is one of the issues identified in our study. The importance of structured communication, which could be a part of standardized reconciliation procedures, is a key factor for the continuity of pharmacological therapy [27,35]. There are some strategies to improve the quality of the reconciliation process, which can be extended to any context. First, the study of Whittington et al. detected an increase of 55% in the accuracy of the reconciliation process by introducing a qualified nursing staff and pharmacist staff [36]. Therefore, the process should be performed in an integrated and multidisciplinary manner to support correct admission and discharge medication reconciliation. Moreover, many studies have reported on the pharmacist as a professional reference in the reconciliation process and have encouraged teamwork among medical doctors, nurses and pharmacists [7,37–39]. Second, a computerized system could facilitate the electronic collection and transfer of medication information from the time of hospital admission to the local care setting discharge, thus reducing the risk of drug discrepancies [3,40–42]. Finally, if the computerization of systems is not possible, a standardized medication reconciliation tool should be introduced to keep track of patient therapy throughout the care transitions [30,43,44].

There are some limitations in our study. First, the reconciliation process is retrospectively assessed. Thus, it was not possible to retrospectively assess whether the drugs involved in the discrepancies were attributable to the fault of the reconnaissance or reconciliation processes; therefore, they were evaluated together. Additionally, it was not possible to measure the potential clinical impact, such as adverse events. Second, the lack of clinical documentation could have limited our analysis because it could not definitively be evaluated whether all cases with a lack of documentation involved medication discrepancies. Third, because of the small sample size, the magnitude of the effect of many of the associations seen cannot be determined with any accuracy. The large size of the confidence intervals seen for major associations indicates that the point estimates for effect size are not likely to be accurate. Finally, the generalizability of our findings could be limited because the results are affected by the type of healthcare system and by the efforts ASLTO2 has made to favor the integration of multiple care settings.

In conclusion, the rate of medication discrepancies across the entire care pathway from hospital admission to a local care setting discharge is low in comparison with results focused on just one care transition. However, this result can be improved, as the local care settings unfortunately have a high lack of communication among professionals with missing documentation, especially in the integrated home care setting. This study suggests that an integrated health care system, such as the Italian system, may influence the prevalence of discrepancies, and highlights the need for structured multidisciplinary and, if possible, computerized medication reconciliation to prevent medication discrepancies and improve the quality of medical documentation.

## Supporting information

S1 Appendix (XLSX)
